# Supplementary figures and images for: Repression of hypoxia-inducible factor-1 contributes to increased mitochondrial reactive oxygen species production in diabetes
Source: eLife. 2022 Feb 15;11:e70714. doi: 10.7554/eLife.70714 (PMC8846593; doi:10.7554/eLife.70714)

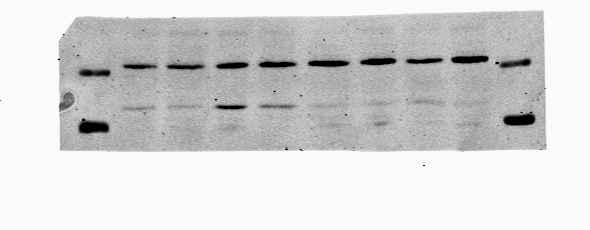

Supplement: Source data 1. [file elife-70714-supp1.zip › unedited blots/Original file_Fig.5E_PHD2_KIM-1&tubulin_unedited blot.tif]

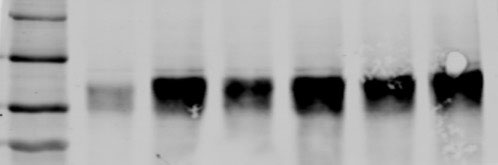

Supplement: Source data 1. [file elife-70714-supp1.zip › unedited blots/Original file_Fig.2A_HIF-1alpha_unedited blot.tif]

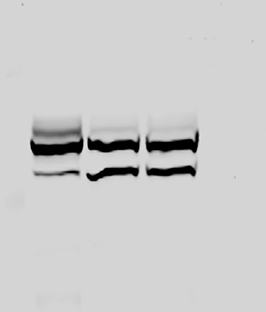

Supplement: Source data 1. [file elife-70714-supp1.zip › unedited blots/Original file_Fig. 5B_dbdb_tubulin_unedited blot.tif]

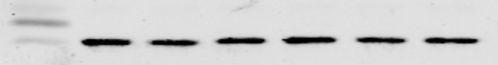

Supplement: Source data 1. [file elife-70714-supp1.zip › unedited blots/Original file_Fig.2A_Histone H3_unedited blot.tif]

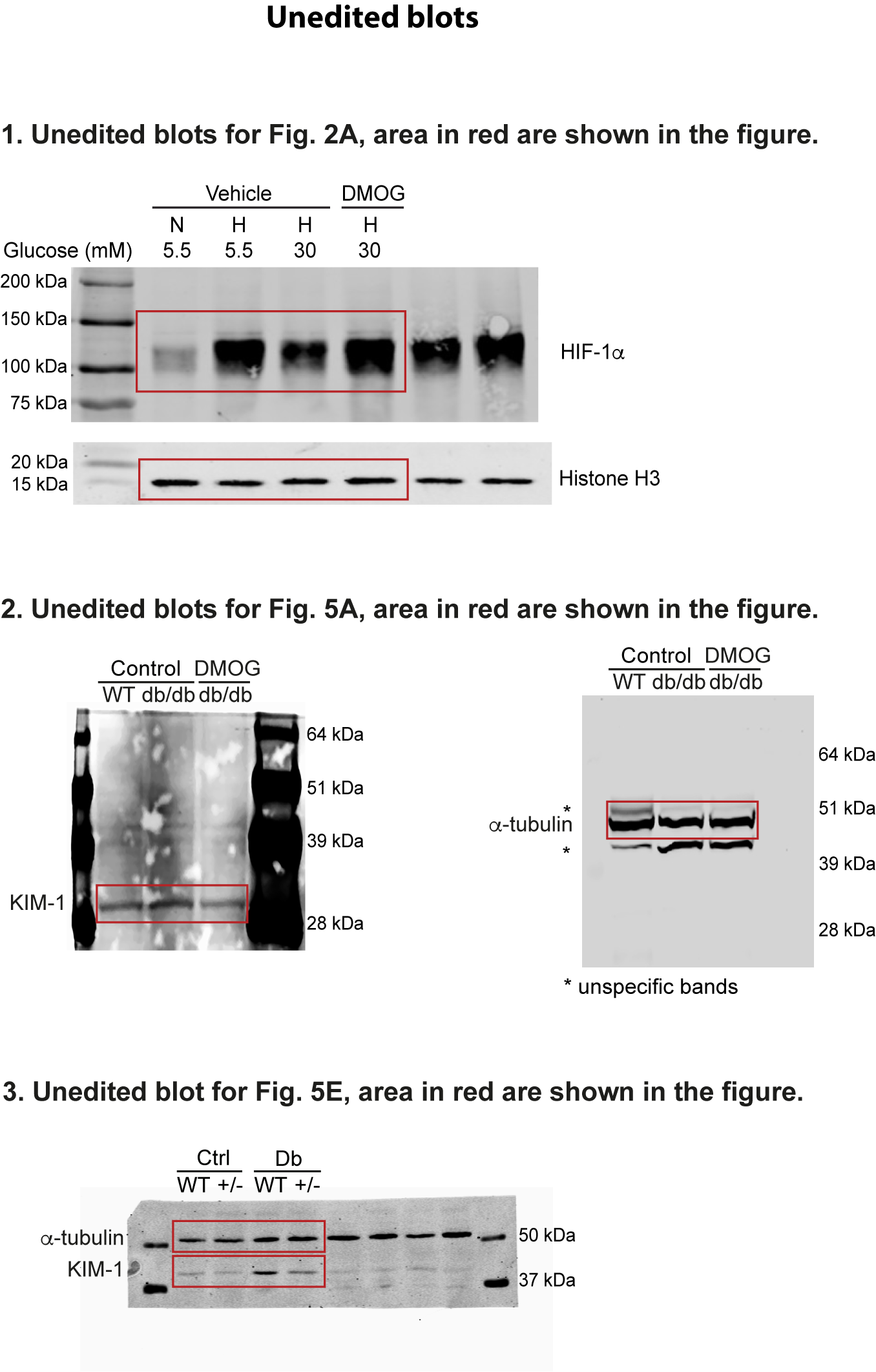

Supplement: Source data 1. [file elife-70714-supp1.zip › unedited blots/Figure with unedited gels.tif]
