# Supplementary material for: Repression of hypoxia-inducible factor-1 contributes to increased mitochondrial reactive oxygen species production in diabetes
Source: eLife. 2022 Feb 15;11:e70714. doi: 10.7554/eLife.70714 (PMC8846593; doi:10.7554/eLife.70714)
Supplement: Transparent reporting form [file elife-70714-transrepform1.pdf]

eLIFE 1st Floor
24 Hills Road
Cambridge CB2 1JP, UK
P 01223 855340
W elifesciences.org
T @elife

eLife's transparent reporting form

We encourage authors to provide detailed information *within their submission* to facilitate the interpretation and replication of experiments. Authors can upload supporting documentation to indicate the use of appropriate reporting guidelines for health-related research (see EQUATOR Network), life science research (see the BioSharing Information Resource), or the ARRIVE guidelines for reporting work involving animal research. Where applicable, authors should refer to any relevant reporting standards documents in this form.

If you have any questions, please consult our Journal Policies and/or contact us: editorial@elifesciences.org.

Sample-size estimation

- You should state whether an appropriate sample size was computed when the study was being designed
- You should state the statistical method of sample size computation and any required assumptions
- If no explicit power analysis was used, you should describe how you decided what sample (replicate) size (number) to use

Please outline where this information can be found within the submission (e.g., sections or figure legends), or explain why this information doesn't apply to your submission:

The sample size of clinical study was decided according to the experience of previous studies. The sample size for animal experiments was calculated to achieve 30% difference in albuminuria between DMOG or vehicle – treated db/db mice or between diabetic PHD2+/- and WT mice and was adjusted for each parameter according to preliminary results. The sample size for in vitro cell experiments was determined using power calculations based on the results from preliminary experiments. This information can be found in the "Materials and Methods" section. The total number of animals or samples in each group of each experiment is denoted in figure legend and each individual data is presented as one dot in the corresponding figure.

Replicates

- You should report how often each experiment was performed
- You should include a definition of biological versus technical replication
- The data obtained should be provided and sufficient information should be provided to indicate the number of independent biological and/or technical replicates
- If you encountered any outliers, you should describe how these were handled
- Criteria for exclusion/inclusion of data should be clearly stated
- High-throughput sequence data should be uploaded before submission, with a private link for reviewers provided (these are available from both GEO and ArrayExpress)

Please outline where this information can be found within the submission (e.g., sections or figure legends), or explain why this information doesn't apply to your submission:

All data used for statistical analysis are independent biological replicates. Technical replicates were applied during luciferase reporter, caspase 3/7 activity, ELISA, and QPCR analysis; and the average of the results from technical replicates is regarded as one biological data. Outliers identified by Grubbs' test were excluded from statistical analysis. This information is included in the "Statistical analysis" section. Inclusion criteria of the mice can be found in the "Animal" section. The number of independent biological replications of each experiment is denoted in figure legend and each individual data is presented as one dot in the corresponding figure.

Statistical reporting

- Statistical analysis methods should be described and justified
- Raw data should be presented in figures whenever informative to do so (typically when N per group is less than 10)
- For each experiment, you should identify the statistical tests used, exact values of N, definitions of center, methods of multiple test correction, and dispersion and precision measures (e.g., mean, median, SD, SEM, confidence intervals; and, for the major substantive results, a measure of effect size (e.g., Pearson's r, Cohen's d)
- Report exact p-values wherever possible alongside the summary statistics and 95% confidence intervals. These should be reported for all key questions and not only when the p-value is less than 0.05.

Please outline where this information can be found within the submission (e.g., sections or figure legends), or explain why this information doesn't apply to your submission:

Statistical analysis method is described in "Statistical analysis" section and also specified in figure legends. Raw data are presented for each figure as individual data points. Sample size and p-values for each experiment are given in figure legends. All data are presented as mean ± SEM as stated in "Statistical analysis" section and figure legends.

(For large datasets, or papers with a very large number of statistical tests, you may upload a single table file with tests, Ns, etc., with reference to sections in the manuscript.)

Group allocation

- Indicate how samples were allocated into experimental groups (in the case of clinical studies, please specify allocation to treatment method); if randomization was used, please also state if restricted randomization was applied
- Indicate if masking was used during group allocation, data collection and/or data analysis

Please outline where this information can be found within the submission (e.g., sections or figure legends), or explain why this information doesn't apply to your submission:

For in vivo experiment, animals were allocated into experimental groups according to their age, blood glucose or HbA1c values. The researchers that performed image analysis of immunohistology and analyzed kidney mitochondrial function were blinded to the identity of the biopsy. This information can be found in "Materials and Methods" section.

Additional data files ("source data")

- We encourage you to upload relevant additional data files, such as numerical data that are represented as a graph in a figure, or as a summary table

eLife Sciences Publications, Ltd is a limited liability non-profit non-stock corporation incorporated in the State of Delaware, USA, with company number 5030732, and is registered in the UK with company number FC030576 and branch number BR015634 at the address 1st Floor, 24 Hills Road, Cambridge CB2 1JP | August 2014

- Where provided, these should be in the most useful format, and they can be uploaded as "Source data" files linked to a main figure or table
- Include model definition files including the full list of parameters used
- Include code used for data analysis (e.g., R, MatLab)
- Avoid stating that data files are "available upon request"

Please indicate the figures or tables for which source data files have been provided:

The source data file contains raw data for all the figures, tables, and the supplementary figures.

eLife Sciences Publications, Ltd is a limited liability non-profit non-stock corporation incorporated in the State of Delaware, USA, with company number 5030732, and is registered in the UK with company number FC030576 and branch number BR015634 at the address 1st Floor, 24 Hills Road, Cambridge CB2 1JP | August 2014
